# Integration of single-cell and bulk RNA sequencing identifies and validates T cell-related prognostic model in hepatocellular carcinoma

**DOI:** 10.1371/journal.pone.0322706

**Published:** 2025-05-02

**Authors:** Yuzhi Zhang, Haiyan Zhang, Lixin Liu

**Affiliations:** 1 Department of Gastroenterology and Hepatology, The First Hospital of Shanxi Medical University, Taiyuan, China; 2 Experimental Center of Science and Research, The First Hospital of Shanxi Medical University, Taiyuan, China; 3 Key Laboratory of Prevention and Treatment of Liver Injury and Digestive System Neoplasms, Provincial Committee of the Medical and Health, Taiyuan, China; Rutgers: Rutgers The State University of New Jersey, UNITED STATES OF AMERICA

## Abstract

Hepatocellular carcinoma (HCC) is a lethal malignancy, and predicting patient prognosis remains a significant challenge in clinical treatment. T cells play a crucial role in the tumor microenvironment, influencing tumorigenesis and progression. In this study, we constructed a T cell-related prognostic model for HCC. Using single-cell RNA sequencing (scRNA-seq) data from the Gene Expression Omnibus (GEO) database, we identified 6,281 T cells from 10 HCC patients and subsequently identified 855 T cell-related genes. Comprehensive analyses were conducted on T cells and their associated genes, including enrichment analysis, cell-cell communication, trajectory analysis, and transcription factor analysis. By integrating scRNA-seq and bulk RNA-seq data with prognostic information from The Cancer Genome Atlas (TCGA), we identified T cell-related prognostic genes and constructed a model using LASSO regression. The model, incorporating PTTG1, LMNB1, SLC38A1, and BATF, was externally validated using the International Cancer Genome Consortium (ICGC) database. It effectively stratified patients into high- and low-risk groups based on risk scores, revealing significant differences in immune cell infiltration between these groups. Differential expression levels of PTTG1 and BATF between HCC and adjacent non-tumor tissues were further validated by immunohistochemistry (IHC) in 25 patient tissue samples. Moreover, a Cox regression analysis was performed to integrate risk scores with clinical features, resulting in a nomogram capable of predicting patient survival probabilities. This study introduces a novel prognostic risk model for HCC patients, aimed at stratifying patients by risk, enhancing personalized treatment strategies, and offering new insights into the role of T cell-related genes in HCC progression.

## 1. Introduction

Hepatocellular carcinoma (HCC) is the most common primary liver cancer, representing approximately 75%-85% of all primary liver cancers. It is also the third leading cause of cancer-related deaths worldwide [1]. HCC typically arises in the context of chronic liver disease and cirrhosis, often associated with hepatitis B or C virus infections, long-term alcohol abuse, and metabolic disorders such as non-alcoholic fatty liver disease [2,3]. Current treatments for HCC include targeted therapies and immunotherapy [4,5]. However, the objective remission rate of HCC mono-immunotherapy is only 15–23%, and the prognosis for HCC is still poor [6,7]. Therefore, there remains an urgent need to predict patient prognosis and to improve immunological efficacy through patient stratification. Ongoing research into the pathogenesis of HCC, identification of new therapeutic targets, and discovery of potential prognostic biomarkers are crucial.

T cells play a critical role in the adaptive immune response to tumors [8]. Their interaction with the tumor microenvironment significantly impacts HCC progression and patient prognosis. For instance, an increase in regulatory T cells (Tregs) within the tumor microenvironment can suppress anti-tumor immune responses, promote tumor growth, and result in poor prognosis [9]. Understanding the dynamics of T cell infiltration and their functional states in HCC can provide valuable insights into potential prognostic biomarkers and therapeutic targets, ultimately improving patient survival and treatment efficacy.

Numerous studies have used RNA sequencing (RNA-seq) data to explore potential prognostic biomarkers for liver cancer. For example, Ji et al. developed a prognostic model for HCC based on genes related to ferroptosis and amino acid metabolism [10], while Xie et al. constructed a prognostic model for alimentary tract malignancies using cuproptosis-related genes [11]. However, bulk RNA-seq can only observe gene changes at the overall level. Single-cell RNA sequencing (scRNA-seq) allows for the analysis of gene expression levels in individual cells within the tumor, identifying distinct cell populations and their specific gene expression profiles [12]. High-resolution data from scRNA-seq plays a crucial role in uncovering the cellular and molecular mechanisms driving tumor progression and immune evasion. Integrating scRNA-seq data with bulk RNA-seq data containing prognostic information allows for constructing robust prognostic models. This approach links cell-specific insights with comprehensive clinical data to predict patient outcomes. In this study, we utilized scRNA-seq to explore cellular heterogeneity within HCC tissues and identified key T cell marker genes. Based on these T cell marker genes, combined with RNA-seq data and clinical information, we constructed a prognostic model for HCC, which includes the genes PTTG1, LMNB1, SLC38A1, and BATF. The stability of the model was validated using external datasets, and we further investigated the model’s relationship with immune infiltration. Our research contributes to the identification of new biomarkers and potential therapeutic targets for HCC.

## 2. Materials and methods

### 2.1. Data download and processing

Single-cell transcriptomes of HCC samples were downloaded from the GEO database (http://www.ncbi.nlm.nih.gov/geo/), specifically GSE149614, which contains 10 HCC samples [13]. Using the “CreateSeuratObject” function of the Seurat package (version 4.3.0.1) in R software (version 4.3.1) [14], the HCC samples were converted into a Seurat object. Cells with 300–7000 features expressed in more than three cells were included. Additionally, the proportion of mitochondrial genes was calculated using the “PercentageFeatureSet” function, and cells with a mitochondrial gene proportion higher than 10% were excluded. After filtering, 31,131 cells remained for further analysis. Bulk RNA sequencing data and associated clinical information for HCC were obtained from TCGA (https://portal.gdc.cancer.gov/) and ICGC (https://dcc.icgc.org/), specifically the Liver Hepatocellular Carcinoma (LIHC) and Liver Cancer - RIKEN, Japan (LIRI-JP) datasets, respectively. The data were subjected to log2 transformation for normalization. All public data were accessed on March 27, 2024.

### 2.2. Data integration and dimensionality reduction

Following quality control, we normalized the data using the ‘NormalizeData’ function in Seurat and identified the top 3000 highly variable genes (HVGs) with the ‘FindVariableFeatures’ function. The “FindIntegrationAnchors” function was used to identify 2000 anchor points, and the “IntegrateData” function was applied to integrated 10 samples based on these anchors to remove batch effects. The integrated data were scaled using the “ScaleData” function, followed by principal component analysis (PCA) on the HVGs using the “RunPCA” function. HCC cell cluster analysis was conducted using the “FindNeighbors” function with the dims parameter set to 1:20, followed by clustering using the “FindClusters” function with the resolution set to 0.8. Visualization was done using t-distributed stochastic neighbor embedding (t-SNE) on a two-dimensional plot.

### 2.3. Cell type identification and DEGs analysis

Cell types were initially annotated using SingleR, with the Human Primary Cell Atlas (HPCA) as the reference dataset. Subsequently, marker genes for each cell type were identified using the “FindAllMarkers” function, applying a logfc threshold of 0.25, the Wilcoxon algorithm, and an adjusted p-value < 0.05. T cells were clustered and annotated into different subtypes based on T cell marker genes reported in the literature. CD4 cells were annotated using CD3D, LTB, and TCF7, while CD8 cells were annotated using CD8A, CD8B, and GZMK [15].

### 2.4. Functional enrichment analysis

To explore the molecular functions of T cell marker genes, functional enrichment analysis was performed using Gene Ontology (GO) and Kyoto Encyclopedia of Genes and Genomes (KEGG) databases as references. Additionally, gene set enrichment analysis (GSEA) was conducted. These analyses were performed using the R packages “clusterProfiler”, “org.Hs.e.g.,db”, and “GSEABase”. A P-value < 0.05 was considered statistically significant, and data visualization was conducted using ggplot2.

### 2.5. Cell-cell communication analysis

To explore interactions between T cells and other cell types, cell-cell communication analysis was performed using the R package “CellChat” [16]. Initially, single-cell RNA sequencing data were input into the CellChat object using the “createCellChat” function. Ligand-receptor pairs were identified, and communication probabilities were calculated using the “computeCommunProb” function, filtering interactions with fewer than three cells (min.cells = 3). Cell-cell communication was inferred at the signaling pathway level and visualized using the “netVisual_bubble” function.

### 2.6. Scenic analysis

SCENIC is a tool for analyzing transcription factors in single-cell RNA sequencing data [17]. We used the SCENIC R package (version 1.3.1) to infer regulatory networks and identify key transcription factors. GENIE3 (version 1.22.0) predicted regulatory relationships between transcription factors and target genes, RcisTarget (version 1.20.0) identified enriched transcription factor binding motifs, and AUCell calculated regulon activity and Regulon Specificity Scores (RSS).

### 2.7. Trajectory analysis

Monocle (v 2.28.0) was used to explore the evolution of T cell subsets [18]. Branched Expression Analysis Modeling (BEAM) was conducted using the Monocle (v 2.28.0) package to analyze gene expression dynamics across different branches of the T cell subset trajectory. T cell data were extracted and analyzed with BEAM to identify branch-specific genes. The identified genes were further subjected to functional enrichment analysis using the GO database. Results were visualized with the “plot_genes_branched_heatmap” function.

### 2.8. Construction of T-related prognostic model

T cell marker genes were intersected with differentially expressed genes (DEGs) from tumor and normal specimens in the TCGA-LIHC dataset (Padj<0.05, logFC = 1), using “DESeq2” package (version 1.40.2) [19]. Expression profiles of the intersected genes were extracted. Combined with patient overall survival (OS), univariate COX regression analysis identified potential prognostic DEGs (P < 0.05). The “glmnet” package was used to perform the least absolute shrinkage and selection operator (LASSO) regression, a method designed to select highly correlated genes and construct a risk model [20]. The risk score for each patient was then calculated using the following formula: Risk score = ∑(Coefficienti × Expressioni), where Coefficienti represents the regression coefficient of the i-th variable, and Expressioni represents the expression level of the i-th variable. Patients were divided into high-risk and low-risk groups using the median risk score as a threshold, a method commonly applied in survival analysis to ensure balanced groups for comparison. The “survival” package generated Kaplan-Meier (K-M) curves. The “timeROC” package plotted the receiver operating characteristic (ROC) curve of the predictive model, and the area under the curve (AUC) value was calculated to evaluate performance for 1, 3, and 5-year OS. Combined with clinical information, the “regplot” package constructed a nomogram, and calibration curves assessed its predictive value.

### 2.9. Validation of the model

The LIRI-JP dataset was used to validate the predictive value of the model. Using the established model, patients in the LIRI-JP dataset were assigned risk scores and grouped into high- and low-risk categories. The model’s performance was evaluated through survival analysis and AUC values, further demonstrating its prognostic reliability.

### 2.10. Nomogram model construction

Clinical variables, including age, sex, and tumor stage, were included in univariate and multivariate Cox regression analyses to assess their prognostic significance. Risk scores, combined with these clinical variables and survival data, were analyzed using the “survival” package. Forest plots generated with the “survminer” and “regplot” packages visualized the independent prognostic value of the risk score. The nomogram incorporating age, sex, stage, and risk scores was constructed using the “rms” package, and calibration curves were generated with the “calibrate” function to evaluate the model’s predictive accuracy.

### 2.11. Immunoinfiltration analysis

TIDE (Tumor Immune Dysfunction and Exclusion) is an analytical tool used to evaluate the response to tumor immunotherapy. It calculates a score predicting patient response to immunotherapy based on T-cell dysfunction and exclusion mechanisms [21]. TIDE scores were obtained from the TIDE website (http://tide.dfci.harvard.edu/) and visualized using the “ggplot2” package. CIBERSORT, based on a deconvolution algorithm, estimates the proportions of different immune cells in bulk RNA-seq data from tumor tissues [22]. CIBERSORT analysis was performed on TCGA-LIHC data using a custom R script (CIBERSORT.R), provided by the original developers of the CIBERSORT algorithm. The script relies on the “e1071” package and the “preprocessCore” package for quantile normalization. Finally, we extracted expression matrices of immune checkpoints from the TCGA-LIHC dataset. Boxplots were utilized to visualize the expression levels of 14 commonly studied immune checkpoints. These immune checkpoints include BTNL9, CD27, CD274, CD276, CD47, CTLA4, HAVCR2, IDO1, LGALS9, PDCD1, TDO2, TIGIT, VSIR, and VTCN1.

### 2.12. Immunohistochemistry (IHC) verification

IHC was used to detect protein expression in HCC patient samples from the First Hospital of Shanxi Medical University. All HCC surgical samples were collected between March 2023 and March 2024. This study was approved by the Ethics Committee of the First Hospital of Shanxi Medical University, and written informed consent was obtained from all participants (protocol number: [2021] K018). Formalin-fixed, dabparaffin-embedded HCC surgical samples were collected and sectioned into 4 μm thick slices. The sections were deparaffinized using xylene and rehydrated through a graded alcohol series. Antigen retrieval was performed by heating the sections in a pressure cooker at 100°C for 3 minutes in Ethylene Diamine Tetraacetic Acid (EDTA) antigen retrieval buffer (pH 9.0) or citrate buffer (pH 6.0). Endogenous peroxidase activity was blocked by incubating the sections with 3% hydrogen peroxide for 20 minutes. Samples were then incubated overnight at 4°C with primary antibodies against BATF (1:100, ER62798, HUABIO, China) and PTTG1 (1:300, JB37–37, HUABIO, China). This was followed by a 30-minute incubation at room temperature with horseradish peroxidase (HRP)-conjugated goat anti-rabbit secondary antibody (PV-9001, ZSGB-BIO, China). Finally, the expression of markers was detected using 3,3’-diaminobenzidine (DAB) staining, counterstained with hematoxylin, dehydrated, and mounted. Quantitative analysis was performed using ImageJ software and data were analyzed using GraphPad Prism software.

### 2.13. Statistical analysis

Statistical analyses were performed using R software (version 4.3.1). Continuous variables were compared using the Wilcoxon Rank-Sum Test. Kaplan–Meier survival curves were evaluated for statistical differences with the Log-Rank Test. Statistical significance was defined as a p-value < 0.05. Results were annotated as follows: * for p < 0.05, ** for p < 0.01, *** for p < 0.001, and “ns” (not significant) for not significant.

## 3. Results

### 3.1. Identification of T cells in the scRNA-seq samples

**Fig 1:**
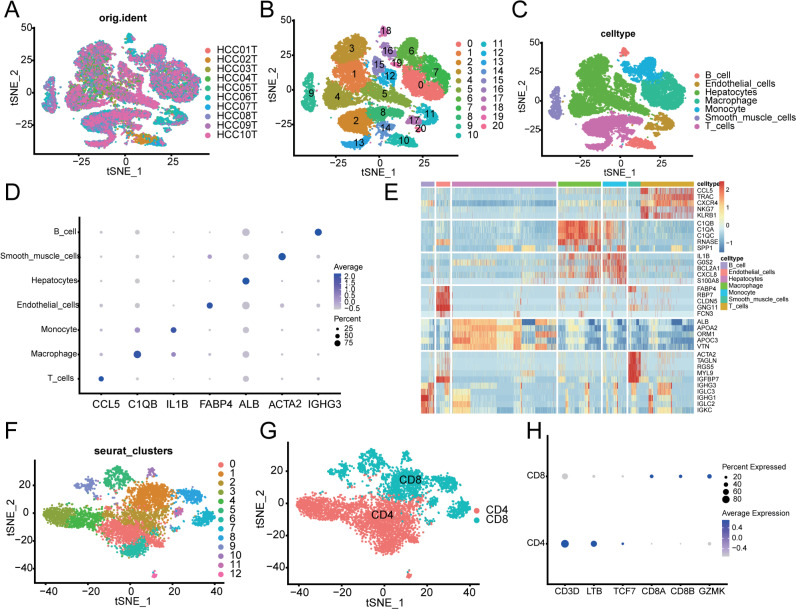
Overview of single cells from 10 HCC samples. (A) t-SNE of HCC samples. (B) t-SNE of the 20 clusters. (C) t-SNE of cell types. (D) Dot plot showing the top 1 marker gene expression across the 7 cell types. (E) Heat map showing the top 5 marker gene expression. (F) t-SNE of 12 clusters. (G) t-SNE of CD4 T cell and CD8 T cell. (H) Dot plot showing cell markers of CD4 and CD8 T cell.

In this study, 10 HCC single-cell samples were analyzed (Fig 1A), and their clinical characteristics are provided in S1 Table. After removing low-quality cells, the samples were normalized and de-batched for subsequent analysis. Dimensionality reduction was performed, and the t-SNE plots displayed the distribution of the 20 clusters (Fig 1B). These 20 clusters were categorized into 7 cell types (Fig 1C). Marker genes for each cell type were identified using a threshold of logFC = 0.25 and adjPval < 0.05, resulting in the identification of 855 T cell marker genes. The top 1 and top 5 marker genes for each cell type were displayed in heatmaps (Fig 1D). Among the cell types, apart from hepatocytes, T cells were the most abundant. Based on marker genes from previous studies, we identified and categorized T cells. Visualization of the t-SNE cluster showed that T cells were divided into 12 clusters and the annotation of CD4 and CD8 cells. (Fig 1F, G). CD4 T cells highly expressed CD3D, LTB, and TCF7, while CD8 T cells highly expressed CD8A, CD8B, and GZMK (Fig 1H).

### 3.2. Comprehensive characterization of T cell functions and regulatory mechanisms in HCC

**Fig 2:**
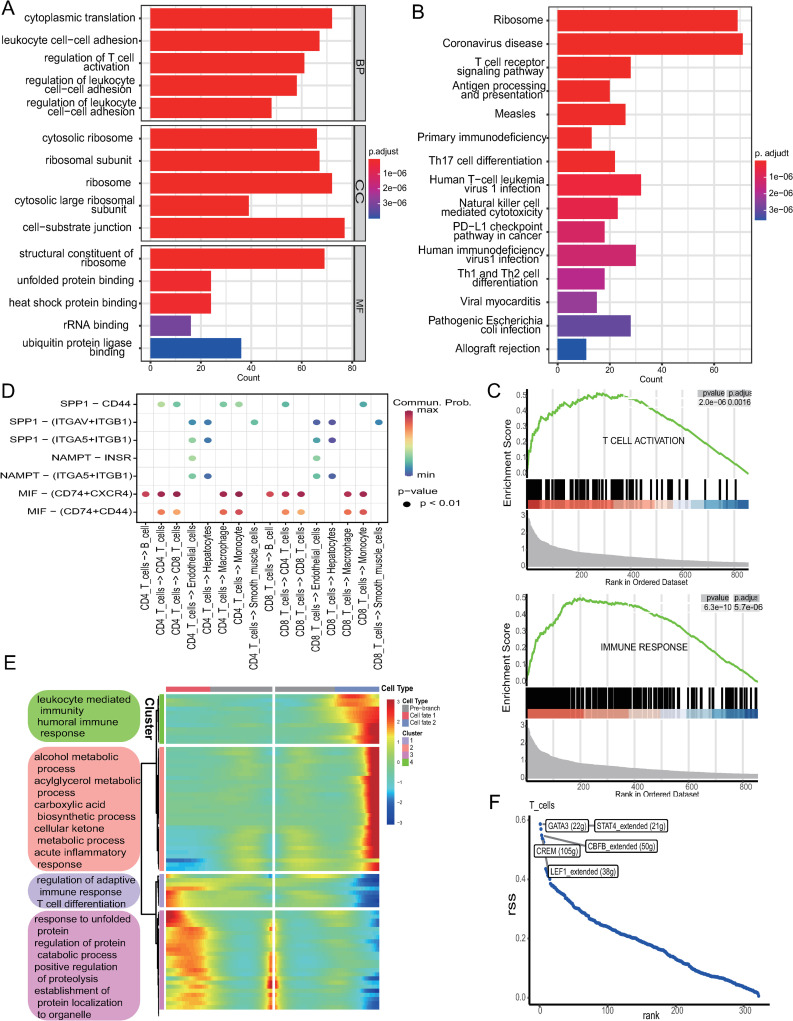
Comprehensive analysis of T cells. (A) GO enrichment analysis of T cells marker genes. (B) KEGG enrichment analysis of T cells marker genes. (C) GSEA enrichment analysis of T cells marker genes. (D) Bubble diagram showing ligand-receptor pair-mediated interactions between T cells and other cells. (E) Differential expressed gene along the pseudotime clustered into four subclusters and the top annotated GO terms in each cluster were provided. (F) SCENIC analysis predicts important TFs in T cell according to RSS.

We performed enrichment analysis on the 855 T cell-related genes identified. GO enrichment analysis revealed that these T cell marker genes were significantly enriched in processes such as regulation of T cell activation, cytosolic ribosome, and structural constituent of ribosome (Fig 2A). KEGG pathway analysis identified significant enrichment in the ribosome, T cell receptor signaling pathway, and Th17 cell differentiation (Fig 2B). Additionally, GSEA analysis demonstrated significant enrichment in T cell activation and immune response pathways (Fig 2C). Cell-cell interaction analysis revealed that the MIF signaling pathway, particularly the MIF-CD74 + CXCR4 axis, was the most prominent contributor to intercellular communication (S1 Fig). Through this axis, as signal senders CD4 and CD8 T cells exhibited notable interactions with B cells, macrophages, and monocytes, highlighting their central role in the tumor immune microenvironment (Fig 2D). Using BEAM analysis, we identified four key branch points along the T cell differentiation trajectory, each associated with unique functional characteristics. Enrichment analysis of branch-specific genes revealed that Cluster 1 was associated with leukocyte-mediated immunity and humoral immune response, Cluster 2 with various metabolic processes, Cluster 3 with regulation of adaptive immune response and T cell differentiation, and Cluster 4 with the regulation of specific proteins (Fig 2E). Finally, transcription factor regulatory network analysis using SCENIC identified GATA3, STAT4, CREM, CBFB, and LEF1 as the top five transcription factors with the highest RSS, underscoring their critical roles in T cell regulation (Fig 2F).

### 3.3. Prognostic model construction and validation

**Fig 3:**
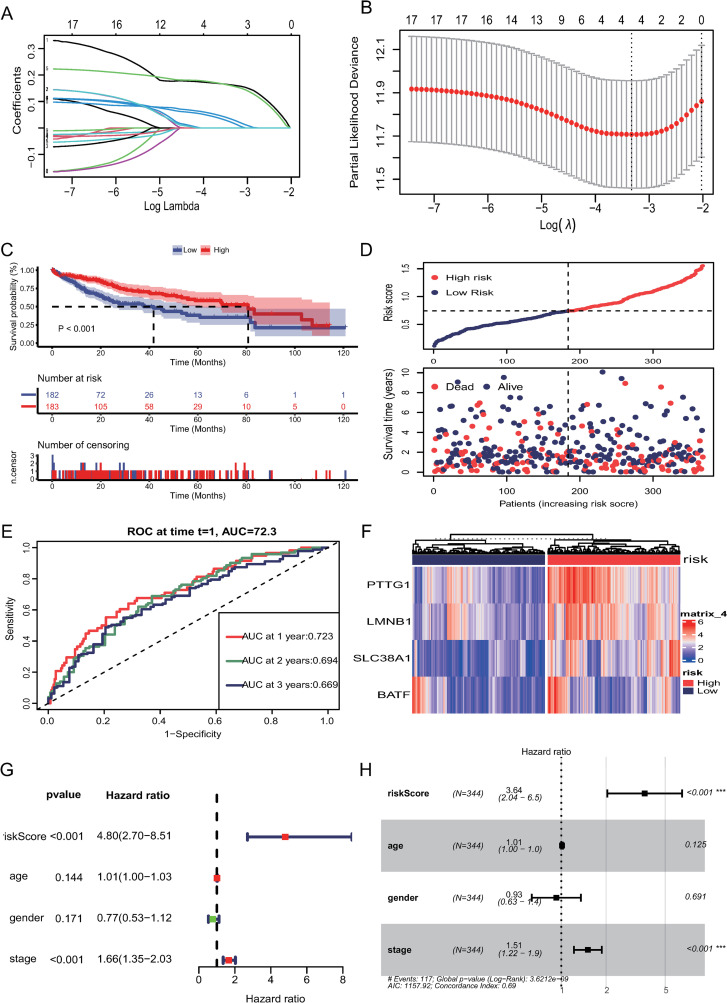
The construction of risk model. (A) Lasso regression identify 4 genes. (B) Cross-validation for the parameters in TCGA dataset. (C) Kaplan-Meier curves of survival analysis in TCGA dataset based on risk score. (D) The distribution of risk score and survival status in TCGA dataset. (E) Time ROC analysis at 1, 2, 3 years in TCGA dataset. (F) Heatmap of identified gene expression in TCGA dataset. (G) Univariate Cox regression analysis revealed the relationship between patients’ overall survival and clinicopathological parameters in TCGA dataset. (H) Multivariate Cox regression analysis in TCGA dataset.

**Fig 4:**
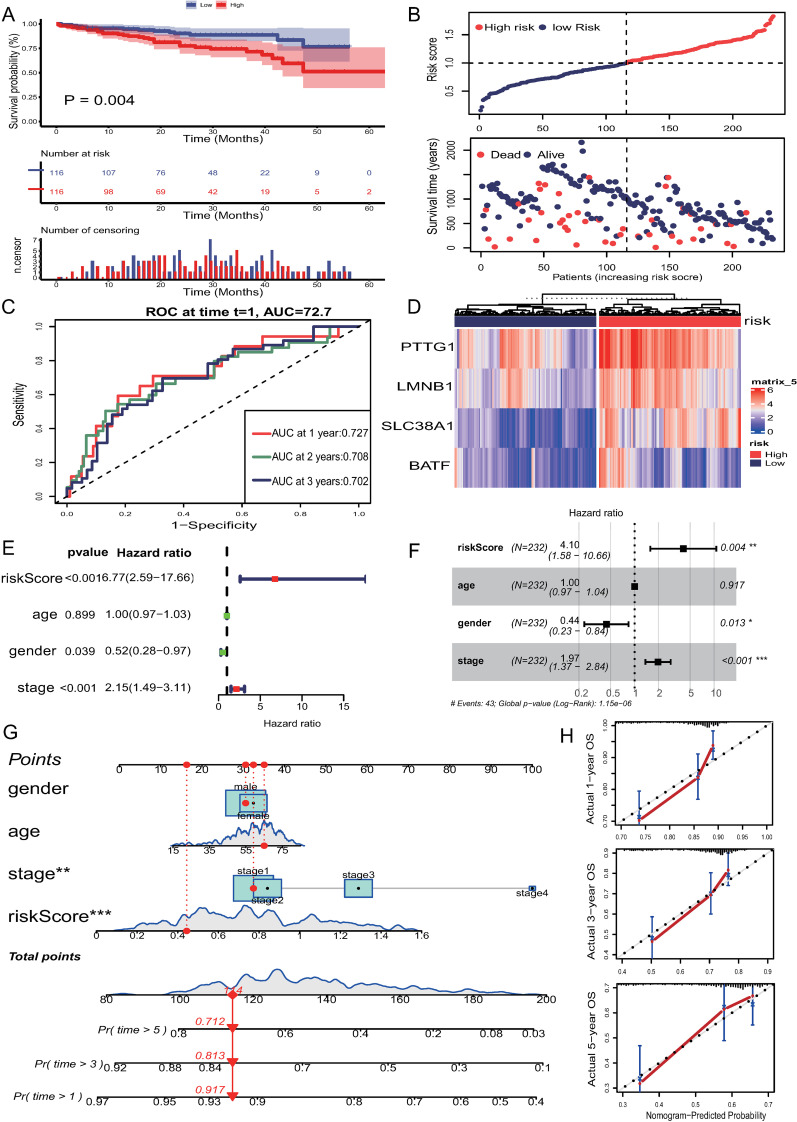
The validation of risk model and construction of nomogram. (A) Kaplan-Meier curves of survival analysis in LIRI-JP dataset based on risk score. (B) The distribution of risk score and survival status in LIRI-JP dataset. (C) Time ROC analysis at 1, 2, 3 years in LIRI-JP dataset. (D) Heatmap of identified gene expression in LIRI-JP dataset. (E) Univariate Cox regression analysis revealed the relationship between patients’ overall survival and clinicopathological parameters in LIRI-JP dataset. (F) Multivariate Cox regression analysis in LIRI-JP dataset. (G) Nomogram depicting the predicted survival rates of patients at 1, 3, and 5 years. (H) The calibration curves for predicting 1, 3, and 5 years.

Table 1 provides an overview of the clinical and pathological characteristics of HCC patients from the TCGA-LIHC and ICGC-LIRI-JP cohorts. The TCGA-LIHC dataset provided mRNA data from 365 tumor tissues and 50 matched normal tissues, along with associated clinical information. Differential analysis on the TCGA-LIHC dataset identified 4504 differentially expressed genes between tumor and normal tissues. These genes intersected with 855 T cell marker genes. The expression matrix of the intersecting genes underwent univariate regression analysis, identifying 17 prognostic genes. LASSO regression analysis ultimately identified 4 genes (Fig 3A, B). The risk score model was defined as: risk score = expression level of PTTG1 * 0.157 + expression level of LMNB1 * 0.0183 + expression level of SLC38A1 * 0.150 + expression level of BATF * 0.005. Patients were classified into high-risk and low-risk groups based on the median risk score. K-M curves indicated that high-risk patients had poorer prognosis (P < 0.001) (Fig 3C, D). Time-dependent ROC curve analysis showed the model effectively predicted overall survival, with AUC values of 0.723, 0.694, and 0.669 for 1-year, 2-year, and 3-year survival, respectively (Fig 3E). The heatmap showed distinct expression patterns of four genes between the high-risk and low-risk groups (Fig 3F). Univariate and multivariate cox regression analysis demonstrated that the risk score was significantly associated with OS and could serve as an independent prognostic factor for LIHC patients (Fig 3G, H). Similar results were observed in the LIRI-JP validation set, where Kaplan-Meier survival analysis revealed that high-risk patients had significantly poorer prognoses (P = 0.04). Additionally, ROC curve analysis showed AUC values of 0.727, 0.708, and 0.702 for 1-, 2-, and 3-year survival predictions, respectively. Furthermore, the forest plot indicated that the risk score serves as an independent prognostic factor (Fig 4A–F).

**Table 1:** Clinical and pathological characteristics of HCC patients in the TCGA-LIHC and ICGC-LIRI-JP cohorts.

	Group information	TCGA: Numbers (percentage)	ICGC: Numbers (percentage)
**sex**	male	246(0.67)	171(0.74)
	female	119(0.33)	61(0.26)
**age**	≤60	173(0.47)	50(0.22)
	>60	192(0.53)	182(0.78)
**stage**	Stage I	171(0.47)	37(0.16)
	Stage II	85(0.23)	104(0.45)
	Stage III	81(0.22)	72(0.31)
	Stage IV	4(0.01)	19(0.08)
	unknow	24(0.07)	0(0.00)
**OS**	Dead	130(0.36)	43(0.18)
	Alive	235(0.64)	189(0.82)

### 3.4. Nomogram construction for survival prediction in HCC patients

To further validate the risk score model, COX regression analysis incorporating clinical information was conducted. Univariate COX regression analysis revealed that the risk score (HR = 4.8, 95% CI = 2.70–8.51, P < 0.001) and stage (HR = 1.66, 95% CI = 1.35–2.03, P < 0.001) significantly affected patient prognosis. Multivariate COX regression analysis confirmed that the risk score (HR = 3.64, 95% CI = 2.04–6.5, P < 0.001) and stage (HR = 1.51, 95% CI = 1.22–1.9, P < 0.001) were independent prognostic factors for overall survival in HCC patients. A nomogram integrating age, gender, stage, and risk score was constructed to predict 1-year, 3-year, and 5-year survival probabilities (Fig 4G). Calibration curves demonstrated good predictive performance, with predicted probabilities closely matching observed outcomes (Fig 4H).

### 3.5. Differential immune landscape and checkpoint expression between high- and low-risk HCC patients

**Fig 5:**
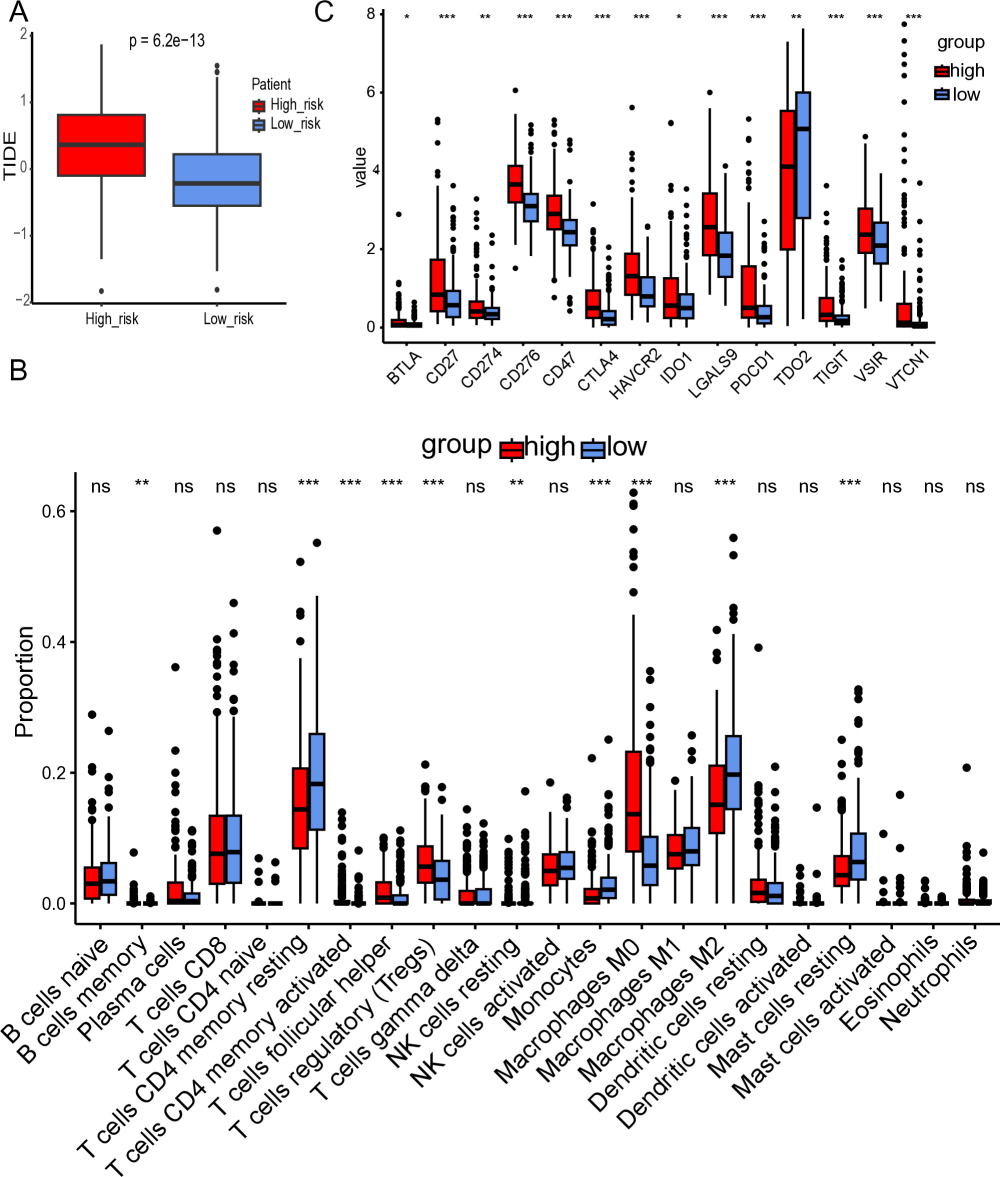
(A) Box plot of TIDE score between high-risk and low-risk groups. (B) Box plot of 16 immune checkpoint level between high-risk and low-risk groups. (C) Box plot of 22 immune cells’ infiltration level between high-risk low-risk groups.

To investigate the relationship between high- and low-risk groups and immune cells, TIDE scores and proportions of 22 immune cell types were calculated for each patient. High TIDE scores indicated significant immune evasion mechanisms and poor response to immune checkpoint inhibitors. Significant differences in TIDE scores were observed between high- and low-risk groups, with high-risk patients having higher TIDE scores and a poorer response to immune checkpoint inhibitors (Fig 5A). CIBERSORT was used to visualize tumor cell proportions for each patient and analyze differences between high- and low-risk groups. High-risk patients had higher proportions of memory B cells, activated CD4 memory T cells, follicular helper T cells, regulatory T cells (Tregs), and M0 macrophages compared to low-risk patients (p < 0.05). Low-risk patients had higher proportions of resting CD4 memory T cells, resting NK cells, monocytes, M2 macrophages, and resting mast cells (p < 0.05) (Fig 5B). Analysis of 14 immune checkpoints showed that, except for BTNL9 and TDO2 which were highly expressed in the low-risk group, all other immune checkpoints were highly expressed in the high-risk group (P < 0.05). Notably, PDCD1 exhibited particularly high expression in the high-risk group. This high expression of immune checkpoints in the high-risk group indicates strong immune evasion capability, associated with high tumor invasiveness and poor prognosis, highlighting the critical role of tumor-infiltrating immune cells in disease progression (Fig 5C).

### 3.6. Validation of prognostic markers using IHC

**Fig 6:**
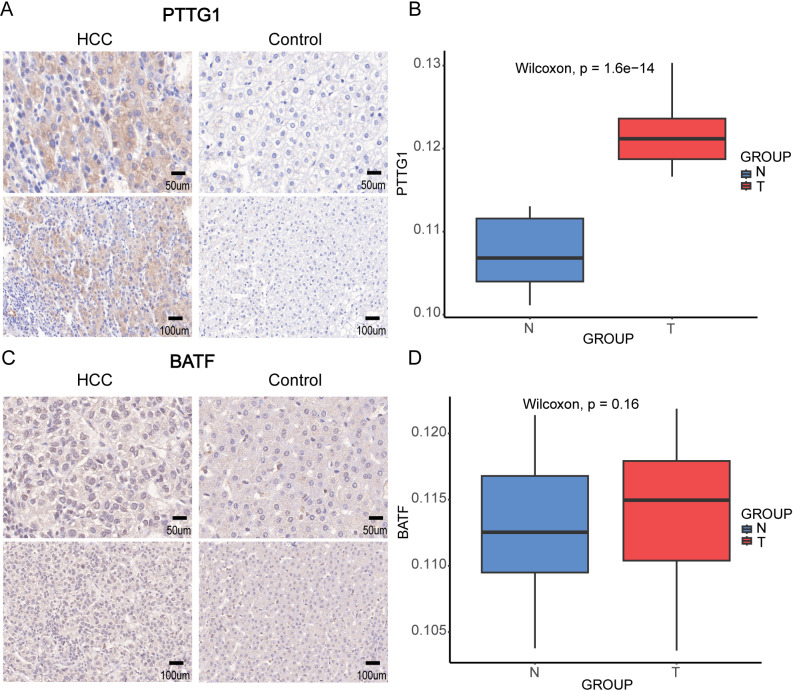
Immunohistochemistry showing the protein expressions of PTTG1 and BATF. (A) The expression of PTTG1 in HCC and Paired non-tumorous tissue. (B) Box plot of PTTG1 expression values. (C) The expression of BATF in HCC and Paired non-tumorous tissue. (D) Box plot of BATF expression values.

To investigate the expression of the PTTG1 and BATF in HCC, we used IHC to examine the expression of PTTG1 and BATF in 25 HCC tissues and their adjacent non-tumorous tissues, and the clinical baseline characteristics of these 25 samples are provided in S2 Table. The results showed significantly higher expression of PTTG1 in tumor tissues compared to adjacent tissues (p < 0.05). PTTG1 was predominantly expressed in both the cytoplasm and nuclei of liver cancer cells, with minor expression observed in the cytoplasm of adjacent tissues (Fig 6A, B). On the other hand, BATF was expressed in both the cytoplasm and nuclei of both tumor and adjacent tissues, with no significant difference in expression between the two groups (p > 0.5, Fig 6C, D).

## 4. Discussion

Hepatocellular carcinoma (HCC) is the sixth most common malignancy worldwide and the fourth leading cause of cancer-related deaths. Despite substantial efforts, the 5-year survival rate remains below 20% [23]. Therefore, new prognostic markers need to be explored. Single-cell sequencing technologies enable gene expression analysis at the individual cell level, providing a more effective interpretation of cellular and tissue heterogeneity [24].This high-resolution molecular approach, combined with bulk RNA-seq data containing prognostic information, facilitates the identification of key biomarkers associated with disease progression and prognosis, thereby enhancing the accuracy of predictive models for clinical outcomes. Moreover, single-cell analysis allows for the examination of immune cells within the tumor microenvironment, revealing the complexity of the tumor immune microenvironment and the functional states and interactions of different immune cell subsets [25]. T cells, a critical component of the adaptive immune system, primarily consist of two cell types: CD4 helper T cells and CD8 cytotoxic T cells [26]. These cells play crucial roles in tumor recognition and anti-tumor responses. Therefore, T cell marker genes are instrumental in identifying potential therapeutic targets and prognostic biomarkers for HCC [27].

This study aimed to explore T cell marker genes and construct a prognostic model for HCC based on these genes. From the literature, we analyzed 10 human liver cancer tissue samples comprising 31,131 cells, revealing seven distinct cell types. T cells constituted the second-largest population after hepatocytes. Enrichment analysis of T cell-related genes indicated associations with cytoplasmic translation and regulation of T cell differentiation within the BP category. Through the identification of marker genes in the literature, T cells were classified into CD4 and CD8 T cell subtypes. CellChat analysis revealed the MIF signaling pathway as the most active in mediating interactions between CD4 and CD8 T cells and other immune cells, including B cells, macrophages, and monocytes, with a particular emphasis on the MIF−(CD74 + CXCR4) axis. MIF, a pro-inflammatory cytokine, plays a crucial role in promoting immune escape and tumor progression within the tumor microenvironment through multiple mechanisms [28,29]. Upregulation of the MIF pathway has been shown to contribute to tumor growth and immune suppression and is associated with poor prognosis in HCC [30,31]. MIF acts as a chemoattractant for B cells, facilitating their recruitment and potentially enhancing antibody-mediated immune responses in collaboration with T cells [32]. Furthermore, macrophages, as major targets of MIF [33], engage in interactions with T cells via MIF signaling, which may amplify inflammatory responses while simultaneously promoting immunosuppression. MIF primarily signals through CD74 and CXCR4(30). The MIF-CD74 may impair the anti-tumor activity of CD8 T cells, promoting tumor proliferation and immune evasion by activating the PI3K-STAT3 and PD-L1 signaling pathways [34]. Additionally, CXCR4 overexpression facilitating epithelial-mesenchymal transition (EMT) in tumors [35,36]. Moreover, the activation of MIF signaling exacerbates the immunosuppressive tumor microenvironment, limiting the activity of effector T cells. Given these roles, targeting the MIF pathway holds potential to improve immune responses and enhance the efficacy of immunotherapy in HCC patients. Time trajectory analysis revealed that genes in T cells change over four distinct developmental stages, each associated with immune response, metabolic processes, T cell differentiation, and protein regulation. SCENIC analysis identified GATA3, STAT4, CBFB, CREM, and LEF1 as the top five transcription factors related to T cells. These transcription factors play a critical role in regulating the development and function of T cells. GATA3 plays a crucial role in balancing immune responses and is a key transcription factor for CD4 T cell development [37]. STAT4 is essential for follicular helper T (Tfh) cell differentiation, and contributes to the production of inflammatory cytokines, promoting the expression of IFN-γ and IL-21, and enhancing cell-mediated immune responses [38]. CBFB regulates T cell homeostasis by modulating the expression of TRIB2 expression during T cell development and function [39]. CREM is involved in the regulation of CD8 expression [40], while LEF1 maintains the suppressive function of regulatory T (Treg) cells, preventing the loss of self-tolerance [41].

HCC prognosis remains poor, necessitating a deeper understanding of how T cell marker genes influence patient outcomes. To address this, we constructed a prognostic risk model based on T cell marker genes using data from TCGA and validated it with the LIRI-JP dataset. The final model includes four key genes: PTTG1, LMNB1, SLC38A1, and BATF, all of which are associated with adverse patient outcomes. PTTG1 (Pituitary Tumor-Transforming Gene 1) encodes securin, a protein crucial for regulating sister chromatid separation during cell division [42]. It is highly expressed in HCC and plays a pivotal role in promoting tumor cell proliferation, migration, invasion, and angiogenesis [43]. Studies have shown that silencing PTTG1 significantly reduces HCC cell proliferation and induces apoptosis, highlighting its potential as a therapeutic target [44]. PTTG1 is involved in multiple oncogenic pathways. Thyroid hormone receptors (TRs) exert tumor-suppressive effects by inhibiting the expression of PTTG1 and the transcription factor Sp1, emphasizing the critical role of the TR-Sp1-PTTG1 signaling axis in HCC progression [45]. Additionally, PTTG1 interacts with β-catenin, stabilizing its nuclear localization and leading to aberrant activation of the Wnt/β-catenin signaling pathway [46]. It also accelerates HCC progression by regulating aspartate metabolism and activating the mTOR signaling pathway, while significantly influencing the tumor microenvironment (TME) [47]. Furthermore, research has demonstrated that Falcarindiol (FAD) enhances HCC sensitivity to the chemotherapeutic agent cisplatin (DDP) by inhibiting the STAT3/PTTG1 signaling pathway [48]. In our study, PTTG1 was identified as a T cell marker gene, suggesting its involvement in immune regulation. It appears to play a dual role in both tumor progression and the shaping of the immune microenvironment. These findings establish PTTG1 as a crucial biomarker and therapeutic target in HCC, offering insights into its potential in both tumor biology and immunotherapy. LMNB1 (Nuclear Lamin B1) is a critical structural protein within the nuclear lamina that provides structural support to the nuclear envelope and maintains cellular stability [49]. In various cancers, including HCC, LMNB1 is highly expressed and plays a significant role in promoting tumor progression. Specifically, LMNB1 enhances cell proliferation, migration, and invasion, partly by disrupting interactions between the nuclear envelope and actin filaments, thereby facilitating cancer cell dissemination [50,51]. In HCC, overexpression of LMNB1 has been associated with advanced pathological staging, poor prognosis, and increased tumor aggressiveness [52]. Furthermore, LMNB1 has been identified as a clinically useful biomarker for early-stage HCC in both tumor tissues and plasma, with its expression levels positively correlating with tumor stage [49,53]. Emerging evidence suggests that LMNB1 also contributes to tumor immune evasion by regulating immune balance within the tumor microenvironment. It is hypothesized to suppress Th1 immune responses while promoting Th2 responses, leading to an imbalance that favors tumor growth and immune escape. Targeting LMNB1 may provide therapeutic benefits by restoring the Th1/Th2 balance and improving patient outcomes, particularly in cancers with LMNB1 overexpression [54]. SLC38A1 (Solute Carrier Family 38 Member 1) is an amino acid transporter that plays a significant role in cancer metabolism. In hepatocellular carcinoma (HCC), SLC38A1 is significantly overexpressed and associated with poor overall survival and recurrence-free survival [55,56]. Its overexpression enhances amino acid uptake, activating the mTORC1 pathway and promoting HCC cell proliferation and survival [57].Conversely, knockdown of SLC38A1 inhibits the viability and proliferation of HCC cells [58]. Additionally, SLC38A1 is essential for glutamine uptake in T cells, linking it to both immune and metabolic functions, making it a potentially interesting target for studying tumor immunity and therapeutic intervention [59,60]. BATF (Basic Leucine Zipper ATF-Like Transcription Factor) is a member of the AP-1/ATF superfamily of transcription factors [61], known for its critical role in regulating T cell differentiation, particularly in regulatory T cells (Tregs) and CD8 + T cells [62,63]. It promotes the development of Tregs, which suppresses anti-tumor immune responses and contributes to a more immunosuppressive tumor microenvironment [64]. Recent studies have identified BATF as a key factor in driving T cell exhaustion, especially in chronic infections and cancer, by activating exhaustion-related genes and inhibits effector T cell functions [65]. In HCC, BATF is highly expressed and associated with poor prognosis. It promotes the formation of immunosuppressive T cells, thereby inhibiting immune responses in HCC. Inhibiting BATF activity may shift the tumor microenvironment from an immunosuppressive state to an immune-activated state, improving patient outcomes [66]. Thus, targeting BATF represents a promising therapeutic strategy to reverse T cell exhaustion and restore immune function in cancers like HCC [61]. Next, we performed immunohistochemical experiments on two genes, PTTG1 and BATF. As PTTG1 had the highest coefficient in our prognostic model, we selected it for IHC analysis. The observed differential expression of PTTG1 in tumors compared to adjacent non-tumor tissues supports its oncogenic role in HCC. BATF plays a key role in T cell exhaustion and tumor immune evasion, processes that are pivotal in cancer progression. However, previous articles have not used IHC to understand BATF expression in HCC. Our IHC results revealed no significant expression differences between tumors and paracancers, which could suggest that BATF’s role in HCC is more related to immune regulation, particularly in T cell exhaustion rather than direct tumor cell activity. This might indicate that BATF contributes to the immunosuppressive environment in HCC, particularly its effects on Tregs and CD8 + T cells.

Using univariate and multivariate analyses, we found that the risk score calculated from our model is an independent prognostic factor for overall survival in HCC patients. Patients were stratified into high-risk and low-risk groups based on their risk scores, we observed significant differences in tumor immune infiltration between the two groups. Specifically, the high-risk group exhibited more severe tumor immune dysfunction and exclusion, confirming their poorer prognoses [67]. Immune cell infiltration revealed that the high-risk group had higher proportions of memory B cells, activated CD4 memory T cells, Tfh, Tregs, and M0 macrophages, whereas the low-risk group displayed higher levels of resting CD4 memory T cells, resting NK cells, monocytes, M2 macrophages, and resting mast cells. Although the activated CD4 memory T cells increased in the high-risk group, their function was likely impaired due to exhaustion within the immunosuppressive tumor microenvironment [68]. Notably, the higher proportion of Tregs in the high-risk group further enhances the immunosuppressive environment by secreting immunosuppressive cytokines and direct cell-cell interactions, inhibiting the anti-tumor activity of T cells [69]. Patients with high infiltration of M0 macrophages in HCC had poorer overall survival, consistent with our findings [70]. The dual roles and complex interactions of immune cells in the tumor microenvironment make it difficult to fully elucidate their relationship with HCC prognosis, warranting further investigation. Checkpoint analysis revealed that PDCD1(PD-1) expression was significantly higher in the high-risk group. PD-1 is often considered a marker for depleted CD8 T cells. The elevated expression of PD-1, in concert with the high proportion of Tregs, significantly inhibits anti-tumor immune responses in the high-risk group [71]. Consequently, immune checkpoint inhibitors targeting PD-1 may prove effective in this subgroup, potentially restoring T cell anti-tumor function. This study enhances our understanding of T cell-associated genes in HCC prognosis but is subject to several limitations. The model helps stratify patients by risk, aiding in the development of personalized treatment strategies and improving survival predictions. However, it was constructed using data from public databases, and its predictive capability needs validation in larger, independent cohorts.

## 5. Conclusions

In summary, we introduced and validated a prognostic model for HCC based on T cell marker genes, emphasizing the roles of PTTG1, BATF, LMNB1, and SLC38A1 in HCC progression. Our immunohistochemistry results confirmed significantly higher expression of PTTG1 in tumor tissues compared to adjacent non-tumorous tissues, reinforcing their role as a major driver of HCC, as indicated by its large coefficient in the model. Conversely, although BATF is recognized for its critical role in T cell exhaustion and immune evasion, we found no significant difference in its expression between tumor and adjacent tissues. This suggests that BATF’s impact in HCC may be more related to immune regulation. Our findings highlight the importance of incorporating immune-related genes in risk stratification for HCC patients. Identifying high-risk groups using this model could potentially guide personalized treatment strategies. However, further validation with larger cohort and mechanistic studies is essential to refine the clinical application of these findings.

## Supporting information

S1 Fig Relative Contribution of Ligand-Receptor Interactions in Signal Pathways. (TIF)

S1 Table Clinical information of single-cell data samples. (DOCX)

S2 Table Clinical information of HCC samples used for IHC. (DOCX)
