# Supplementary figures and images for: Integration of single-cell and bulk RNA sequencing identifies and validates T cell-related prognostic model in hepatocellular carcinoma
Source: PLoS One. 2025 May 2;20(5):e0322706. doi: 10.1371/journal.pone.0322706 (PMC12047759; doi:10.1371/journal.pone.0322706)

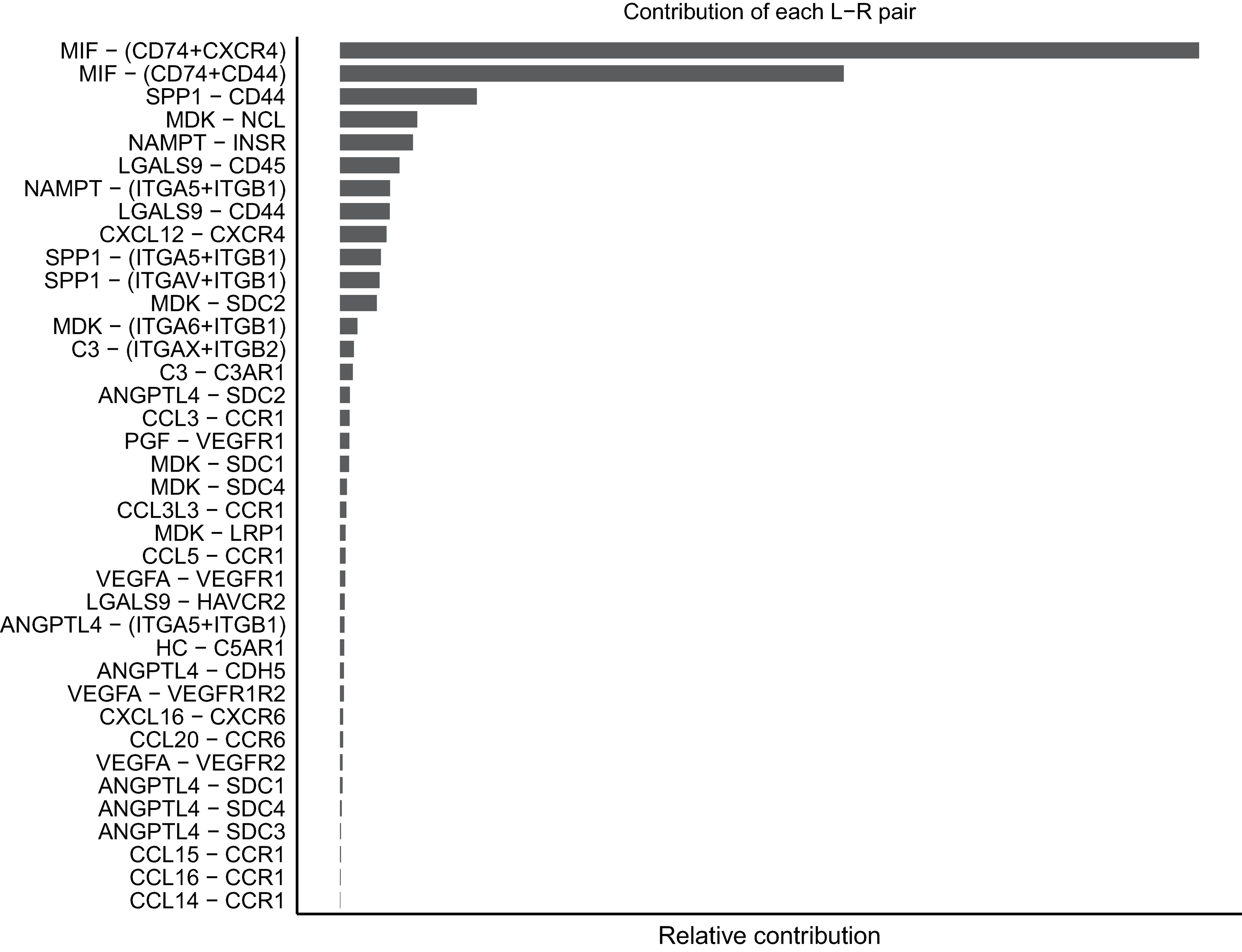

Supplement: S1 Fig — (TIF) [file pone.0322706.s001.tif]
