# Supplementary material for: Integration of single-cell and bulk RNA sequencing identifies and validates T cell-related prognostic model in hepatocellular carcinoma
Source: PLoS One. 2025 May 2;20(5):e0322706. doi: 10.1371/journal.pone.0322706 (PMC12047759; doi:10.1371/journal.pone.0322706)
Supplement: S1 Table — (DOCX) [file pone.0322706.s002.docx]

| **Patient ID** | **Gender** | **Age** | **Virus infection** | **T** | **N** | **M** | **stage** |
| --- | --- | --- | --- | --- | --- | --- | --- |
| **HCC01** | Male | 66 | HBV | T1 | N0 | M0 | I |
| **HCC02** | Male | 65 | HBV | T1 | N0 | M0 | I |
| **HCC03** | Male | 66 | HCV | T1 | N0 | M0 | I |
| **HCC04** | Male | 60 | HCV | T2 | N0 | M0 | II |
| **HCC05** | Male | 65 | None | T3 | N0 | M0 | IIIA |
| **HCC06** | Female | 64 | HBV | T3 | N0 | M0 | IIIA |
| **HCC07** | Male | 48 | None | T4 | N0 | M0 | IIIB |
| **HCC08** | Male | 64 | None | T4 | N0 | M0 | IIIB |
| **HCC09** | Male | 48 | HBV | T4 | N0 | M0 | IV |
| **HCC10** | Male | 53 | HBV | T4 | N0 | M1 | IV |

**S1 Table.** Clinical information of single-cell data samples.
