# Supplementary material for: Integration of single-cell and bulk RNA sequencing identifies and validates T cell-related prognostic model in hepatocellular carcinoma
Source: PLoS One. 2025 May 2;20(5):e0322706. doi: 10.1371/journal.pone.0322706 (PMC12047759; doi:10.1371/journal.pone.0322706)
Supplement: S2 Table — (DOCX) [file pone.0322706.s003.docx]

|  | **sex** | **age** | **Virus infection** | **T** | **N** | **M** | **stage** | **ALT** | **AST** | **Albumin** | **Prothrombin Time** |
| --- | --- | --- | --- | --- | --- | --- | --- | --- | --- | --- | --- |
| 1 | Male | 78 | None | T2 | N0 | M0 | Ⅱ | 38 | 37 | 39.4 | 15.6 |
| 2 | Male | 51 | HCV | T2 | N0 | M0 | Ⅱ | 15 | 17 | 43.8 | 13.2 |
| 3 | Male | 47 | HBV | T3 | N1 | M0 | ⅣA | 322 | 285 | 35.7 | 14.7 |
| 4 | Male | 51 | HBV | T2 | N0 | M0 | Ⅱ | 153 | 58 | 32.9 | 12.4 |
| 5 | Male | 55 | HBV | T1a | N0 | M0 | ⅠA | 10 | 21 | 42.4 | 14 |
| 6 | Male | 65 | HBV | T4 | N0 | M0 | ⅢB | 986 | 1668 | 38.1 | 14.9 |
| 7 | Female | 71 | HBV | T1b | N0 | M0 | ⅠB | 38 | 42 | 39.9 | 11.9 |
| 8 | Male | 50 | HBV | T1a | N0 | M0 | ⅠA | 181 | 58 | 36.3 | 13.6 |
| 9 | Male | 59 | None | T3 | N0 | M0 | ⅢA | 159 | 248 | 34.8 | 14.3 |
| 10 | Male | 62 | HBV | T2 | N0 | M0 | Ⅱ | 54 | 62 | 36.8 | 11.6 |
| 11 | Female | 59 | None | T3 | N0 | M0 | ⅢA | 22 | 14 | 32.6 | 14.1 |
| 12 | Female | 50 | None | T1b | N0 | M0 | ⅠB | 9 | 21 | 38.2 | 13.4 |
| 13 | Male | 30 | None | T3 | N0 | M0 | ⅢA | 256 | 318 | 47.9 | 13.7 |
| 14 | Female | 47 | HBV | T3 | N0 | M0 | ⅢA | 47 | 91 | 38.7 | 12.2 |
| 15 | Male | 60 | HBV | T2 | N0 | M0 | Ⅱ | 17 | 33 | 26.2 | 17.9 |
| 16 | Female | 51 | HBV | T3 | N0 | M0 | ⅢA | 14 | 29 | 33.9 | 15.2 |
| 17 | Male | 61 | None | T3 | N0 | M0 | ⅢA | 96 | 126 | 26 | 12.5 |
| 18 | Male | 55 | HBV | T3 | N0 | M0 | ⅢA | 60 | 58 | 43.8 | 11.6 |
| 19 | Female | 64 | HBV | T2 | N0 | M0 | Ⅱ | 18 | 32 | 38.7 | 15.0 |
| 20 | Male | 54 | None | T1b | N0 | M0 | ⅠB | 24 | 26 | 38.8 | 12.4 |
| 21 | Female | 69 | None | T1a | N0 | M0 | ⅠA | 638 | 1142 | 26.5 | 13.8 |
| 22 | Male | 52 | HBV | T1b | N0 | M0 | ⅠB | 33 | 29 | 42.1 | 11.6 |
| 23 | Male | 61 | HBV | T1b | N0 | M0 | ⅠB | 14 | 17 | 39.2 | 13.2 |
| 24 | Male | 55 | HBV | T4 | N0 | M0 | ⅢB | 318 | 222 | 35.1 | 23.2 |
| 25 | Female | 61 | HBV | T4 | N0 | M0 | ⅢB | 11 | 20 | 43.6 | 14.4 |

**S2 Table.** Clinical information of HCC samples used for IHC.
